# An Analytic Model of Transient Heat Conduction for Bi-Layered Flexible Electronic Heaters by Symplectic Superposition

**DOI:** 10.3390/mi13101627

**Published:** 2022-09-28

**Authors:** Dian Xu, Sijun Xiong, Fanxing Meng, Bo Wang, Rui Li

**Affiliations:** 1 State Key Laboratory of Structural Analysis for Industrial Equipment, Department of Engineering Mechanics, International Research Center for Computational Mechanics, Dalian University of Technology, Dalian 116024, China; 2 Department of Strength, AVIC Shenyang Aircraft Design and Research Institute, Shenyang 110035, China

**Keywords:** flexible electronic heater, heat conduction, symplectic superposition, analytic solution

## Abstract

In a flexible electronic heater (FEH), periodic metal wires are often encapsulated into the soft elastic substrate as heat sources. It is of great significance to develop analytic models on transient heat conduction of such an FEH in order to provide a rapid analysis and preliminary designs based on a rapid parameter analysis. In this study, an analytic model of transient heat conduction for bi-layered FEHs is proposed, which is solved by a novel symplectic superposition method (SSM). In the Laplace transform domain, the Hamiltonian system-based governing equation for transient heat conduction is introduced, and the mathematical techniques incorporating the separation of variables and symplectic eigen expansion are manipulated to yield the temperature solutions of two subproblems, which is followed by superposition for the temperature solution of the general problem. The Laplace inversion gives the eventual temperature solution in the time domain. Comprehensive time-dependent temperatures by the SSM are presented in tables and figures for benchmark use, which agree well with their counterparts by the finite element method. A parameter analysis on the influence of the thermal conductivity ratio is also studied. The exceptional merit of the SSM is on a direct rigorous derivation without any assumption/predetermination of solution forms, and thus, the method may be extended to more heat conduction problems of FEHs with more complex structures.

## 1. Introduction

Electric heating devices have attracted scholarly interest due to their important engineering and medical applications during the past few decades, including resistive micro heaters [1], electrothermal pumping devices [2], shape memory polymer micro actuators for drug delivery [3], homoeothermic maintenance in small animals [4], etc. However, they are designed within rigid structures that cannot heat undevelopable curved surfaces, leading to inhomogeneous temperature distributions caused by inevitable wrinkles from non-developable designs. To resolve this problem, recent advancements in flexible electronic heaters (FEHs) have arisen.

The FEHs developed in recent years can be divided into two major categories. One is on polymers with a variety of conductive compositions, including transparent copper fiber heaters [5], metal nanowire heaters for wearable electronics applications [6], organic conductive polymers [7], carbon nanotube-based resistive heater textile [8], stretchable tattoo-like heaters with on-site temperature feedback control [9], etc. Under stretching circumstances, the electrical resistance of an FEH will increase rapidly due to the deformation of the fibers, thus leading to extraordinarily non-uniform temperatures, i.e., hot spots [5]. In addition, extremely complicated manufacturing techniques are required to produce such FEHs. The other category is encapsulating periodic metal wires into the soft elastic substrate performing as heat sources to avoid hot spots, such as kirigami-patterned wearable thermotherapy devices [10], two-dimensional serpentine configured electronics [11], three-dimensional network devices [12], multifunctional membranes in cardiac electrotherapy [13], etc. In this way, the electrical resistance of periodic metal wires will behave stably due to their flexible deformation. However, designable heat sources demand spaces to keep deformability and extensibility, resulting in different temperatures at certain positions. Since the localized high temperature will burn the tissue and it is extraordinarily hard to control the heating power, temperature uniformity is usually a key point of FEHs in engineering applications. 

Great efforts have been made to achieve temperature uniformity in FEHs via periodic wire structures embedded into substrate layers. Various conductive wire heaters, which are composed of metallic nanowires [14], carbon nanotubes [15], conductive polymers [16], or hybrid materials [17], have been studied for realizing the optimal performances of the FEHs. Comparatively, metallic nanowires, e.g., Ag nanowires, not only have outstanding conductivity but also can be obtained via an uncomplicated preparation process with low cost, resulting in wide applications in FEHs. In addition to experimental studies, it is necessary to develop analytic models on the transient heat conduction of FEHs. In this way, the time-dependent temperature distribution can be rapidly predicted, and the effects of key parameters can be rapidly analyzed, which is very useful for the layout design of wire structures in FEHs. However, given the previously reported results, there have been very few studies on the analytic modeling of transient heat conduction for FEHs.

**Figure 1:**
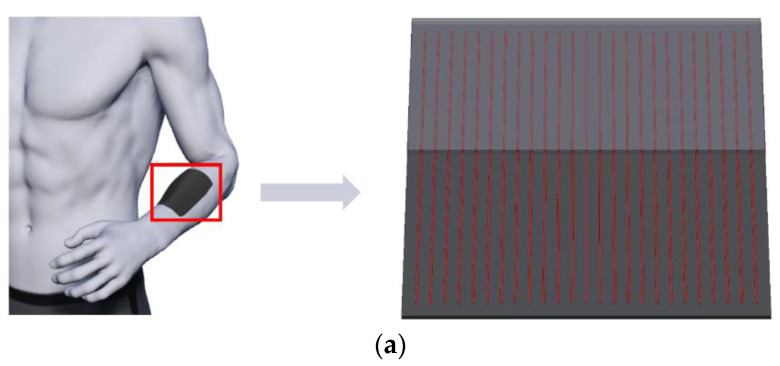

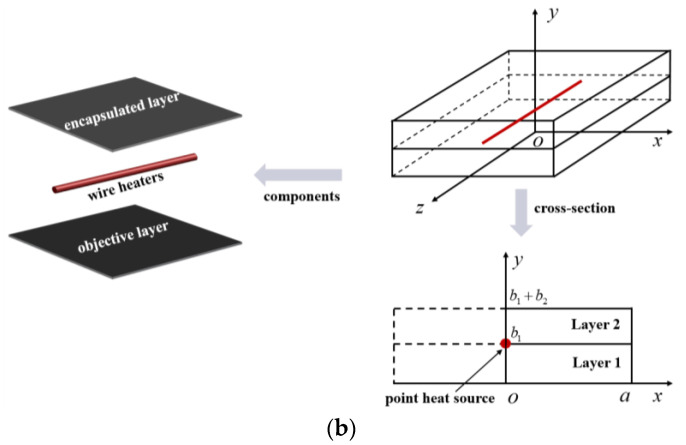
Schematic illustration of (**a**) a bi-layered FEH with parallel wire structures integrated with human skin and (**b**) a bi-layered transient heat conduction model.

Figure 1a shows the schematic illustration of an easy-to-implement bi-layered FEH, where parallel wire heaters are sandwiched between an objective layer and an encapsulated layer. The transient heat conduction problem of such a structure is first equivalent to that of a single component due to the periodicity (Figure 1b, left and upper right). It is further simplified to a two-dimensional problem by taking a cross-section that is perpendicular to the wire in the component (Figure 1b, lower). Details appear in Section 2. The major difficulty hindering the analytic modeling of such a bi-layered transient heat conduction model is in solving the governing partial differential equations (PDEs) incorporating prescribed temperature/heat flux boundary conditions (BCs).

**Figure 2:**
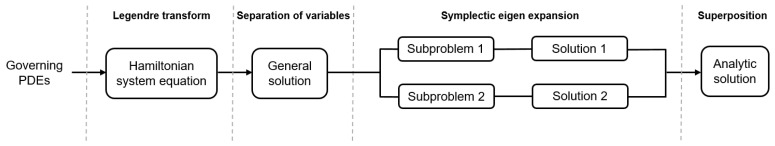
Flowchart of the solution procedure for the SSM.

In recent years, we have proposed a novel analytic symplectic superposition method (SSM) that has been successfully applied to bending [18], buckling [19], and vibration [20] of plate and shell structures as well as the mechanical analysis of flexible electronics [21]. The method involves a skillful combination of the superposition method and the symplectic approach [22], which is conducted within the Hamiltonian framework in the symplectic space rather than within the Lagrangian system in the Euclidean space where the conventional analytic methods are conducted. The main idea of the SSM (Figure 2) is to transform the governing PDEs of a problem into the Hamiltonian system to establish the subproblems that can be analytically solved by the symplectic approach, and the eventual analytic solution is obtained according to the equivalence between the general problem and the superposition of the subproblems. 

Compared with the conventional methods, the exceptional merit of SSM is the inherent rigorous derivation without any assumption and pre-determination of solution forms, which enables it to serve as a general method for new analytic solutions. Although the main applications of the SSM have been on the mechanical problems of structures, it may find access to some new issues that are physically different from the mechanical problems.

In this study, we make an SSM-based successful attempt to achieve the transient heat conduction analysis for bi-layered FEHs. The governing equation in the Hamiltonian system is introduced in Section 2, where the symplectic eigenproblems are generated by the separation of variables in the symplectic space. In Section 3, the basic solution procedure of subproblems is presented, and the eventual solution is obtained by superposition. The convergent time-dependent temperature results obtained by the SSM are given in Section 4, with the verification by the finite element method (FEM). With the transient solution, the steady-state results are also revealed, and the effect of the thermal conductivity ratio is discussed. Conclusions are drawn in Section 5.

## 2. Governing Equation for Bi-Layered Transient Heat Conduction within the Hamiltonian Framework

As is shown in Figure 1b, lower, a two-dimensional half model is considered for a single component due to the symmetry with respect to the *oy* axis, with the entire dimensions along *ox* and *oy* axes being a and $b_{1}+b_{2}$, respectively. The thickness of Layer 1 (the bottom objective layer) is $b_{1}$ and that of Layer 2 (the top encapsulated layer) is $b_{2}$. The wire heat source is modeled as a point heat source (with a half heat source intensity) in the cross-section, as indicated by the semicircular red dot at $\left(0,b_{1}\right)$. The governing transient heat conduction equation is written as [23]

(1) $$\frac{1}{\alpha_{i}}\frac{\partial T_{i}\left(x,y,t\right)}{\partial t}=\frac{\partial^{2}T_{i}\left(x,y,t\right)}{\partial x^{2}}+\frac{\partial^{2}T_{i}\left(x,y,t\right)}{\partial y^{2}}+\frac{1}{k_{i}}g_{i}\left(x,y,t\right)$$

Here, the quantities with a subscript i (=1 or 2) represent those for Layer i. $T_{i}\left(x,y,t\right)$ is the temperature increment function, $g_{i}\left(x,y,t\right)$ is the heat source, t is time, $\alpha_{i}=k_{i}/\left(\rho_{i}c_{i}\right)$ is the thermal diffusivity with $k_{i}$ being the thermal conductivity, $\rho_{i}$ being the density, and $c_{i}$ being the specific heat capacity.

The BCs at the upper and lower surfaces yield

(2) $${\left(\kappa_{i}\nabla T_{i}+\eta_{i}T_{i}\right)|}_{y=0,b_{1}+b_{2}}=\gamma_{i}\left(x,y,t\right)$$

In this study, it comes to either Dirichlet type, with $\kappa_{i}=0$ and $\eta_{i}\neq 0$, or Neumann type, with $\kappa_{i}\neq 0$ and $\eta_{i}=0$. The left and right sides are subjected to adiabatic BCs due to the symmetry with respect to the *oy* axis and the periodicity along the *ox* axis, respectively.

The Laplace transform of a real function f:ℝ→ℝ, with f(t)=0 for t<0, and its inversion are defined as [24]

(3) $$\begin{array}{c} F\left(s\right)=L\left[f\left(t\right)\right]=\int_{0}^{\infty }e^{-st}f\left(t\right)\;dt\\ f\left(t\right)=L^{-1}\left[F\left(s\right)\right]=\frac{1}{2\pi i}\int_{v-I\infty }^{v+I\infty }e^{st}F\left(s\right)\;ds \end{array}$$

with s=v+Iw (v,w∈ℝ), where I is the imaginary unit. v∈ℝ is arbitrary but greater than the real parts of all the singularities of F(s). 

The governing equation and BCs are converted into the Laplace transform domain:

(4) $$\begin{array}{l} \frac{s{\bar{T}}_{i}\left(x,y,s\right)}{\alpha_{i}}=\frac{\partial^{2}{\bar{T}}_{i}\left(x,y,s\right)}{\partial x^{2}}+\frac{\partial^{2}{\bar{T}}_{i}\left(x,y,s\right)}{\partial y^{2}}+\frac{1}{k_{i}}{\bar{g}}_{i}\left(x,y,s\right)\\ \kappa_{i}\nabla {\bar{T}}_{i}+\eta_{i}{\bar{T}}_{i}={\bar{\gamma }}_{i}\left(x,y,s\right) \end{array}$$

where the variables with an overbar represent those in the Laplace transform domain and *s* represents the Laplace transform parameter. Introduce the Lagrangian function [25]:

(5) $$L_{i}=\frac{k_{i}}{2}\left[{\dot{\bar{T}}}_{i}{}^{2}+{\left(\frac{\partial {\bar{T}}_{i}}{\partial x}\right)}^{2}+\frac{s{\bar{T}}_{i}{}^{2}}{\alpha_{i}}\right]-{\bar{g}}_{i}{\bar{T}}_{i}$$

where the over dot indicates the partial derivative about *y*. Based on the principle of the least action for the transient heat conduction process [26], we acquire

(6) $$\delta \int \int L_{i}dxdy=0$$

It is noted that for the transient heat conduction process, the entransy dissipation rate closely connects to the integral of the convolution of heat flux and negative temperature gradient over the time and space domain, whose convolution integral consists of the influence of the time evolution. In addition, the transient heat conduction process includes both the dissipating and non-dissipating processes, and thus, its variational function could include both the dissipating and non-dissipating terms [27,28], as depicted in Equation (5). 

Defining

(7) $${\bar{q}}_{i}={\bar{T}}_{i}$$

its dual variable can be obtained by Legendre transform, yielding

(8) $${\bar{p}}_{i}=\frac{\partial L_{i}}{\partial {\dot{T}}_{i}}=k_{i}\frac{\partial {\bar{T}}_{i}}{\partial y}$$

The Hamiltonian function is introduced according to Equations (7) and (8) as

(9) $$H_{i}\left({\bar{q}}_{i},{\bar{p}}_{i}\right)={\bar{p}}_{i}{\dot{\bar{q}}}_{i}-L_{i}$$

with ${\dot{\bar{q}}}_{i}=\partial H_{i}/\partial {\dot{\bar{p}}}_{i}$ and ${\dot{\bar{p}}}_{i}=-\partial H_{i}/\partial {\bar{q}}_{i}-{\bar{f}}_{i}$. Accordingly, the variation of Equation (9), $\delta H_{i}\left({\bar{q}}_{i},{\bar{p}}_{i}\right)=0$ yields the following Hamiltonian-system equation

(10) $${\dot{Z}}_{i}=H_{i}Z_{i}+f_{i}$$

where $Z_{i}={\left[{\bar{q}}_{i},{\bar{p}}_{i}\right]}^{T}$ is the state vector, $f_{i}={\left[0,-{\bar{g}}_{i}\right]}^{T}$ is the vector concerning the heat source, and ${\bar{g}}_{i}=\left(Q_{i}/s\right)\delta \left(x-x_{i}\right)\delta \left(y-y_{i}\right)$ is the point heat source with a constant heat source intensity $Q_{i}$ and the Dirac delta function δ.

(11) $$H_{i}=\left[\begin{array}{cc} 0 & \frac{1}{k_{i}}\\ \frac{sk_{i}}{\alpha_{i}}-k_{i}\frac{\partial^{2}}{\partial x^{2}} & 0 \end{array}\right]$$

is the Hamiltonian operator matrix that satisfies $H_{i}^{T}=JH_{i}J$, where $J=\left[\begin{array}{cc} 0 & 1\\ -1 & 0 \end{array}\right]$ is a symplectic matrix [22].

Separating the variables in the symplectic space as $Z_{i}=X^{i}\left(x\right)Y^{i}\left(y\right)$, where $X^{i}\left(x\right)={\left[{\bar{q}}_{i}\left(x\right),{\bar{p}}_{i}\left(x\right)\right]}^{T}$ is a vector depending only on *x*, and $Y^{i}\left(y\right)$ is a function depending only on *y*, we have

(12) $$\begin{array}{c} \frac{dY^{i}\left(y\right)}{dy}=\mu Y^{i}\left(y\right)\\ H_{i}X^{i}\left(x\right)=\mu X^{i}\left(x\right) \end{array}$$

where μ is a non-zero eigenvalue, and $X^{i}\left(x\right)$ is the corresponding eigenvector. The characteristic equation of the second half of Equation (12) is

(13) $${\left(\lambda_{i}\right)}^{2}+{\left(\mu^{i}\right)}^{2}=\frac{s}{\alpha_{i}}$$

with the roots

(14) $$\lambda_{i}=\pm I\beta^{i}$$

and

(15) $$\beta^{i}=\sqrt{{\left(\mu^{i}\right)}^{2}-\;\frac{s}{\alpha_{i}}}$$

The general temperature solution in the Laplace transform domain is therefore

(16) $${\bar{T}}_{i}\left(x\right)=A_{i}\cos\left(\beta^{i}x\right)+B_{i}\sin\left(\beta^{i}x\right)$$

where $A_{i}$ and $B_{i}$ are coefficients undetermined hitherto.

## 3. New Analytic Solution by the SSM

**Figure 3:**
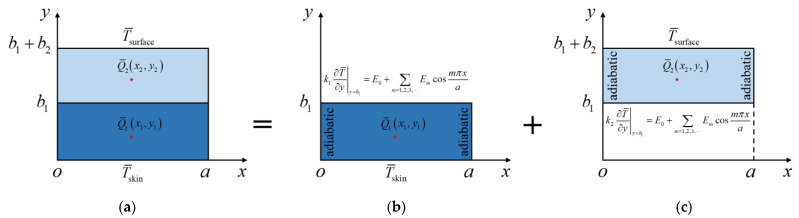
SSM procedure for bi-layered transient heat conduction with two arbitrarily positioned point heat sources. (**a**) General problem. (**b**) Subproblem for Layer 1. (**c**) Subproblem for Layer 2.

Since the main purpose of this study is to provide an SSM-based analytic model of transient heat conduction for bi-layered FEHs, a general problem with an arbitrarily positioned point heat source at $\left(x_{1},y_{1}\right)$ in Layer 1 and an arbitrarily positioned point heat source at $\left(x_{2},y_{2}\right)$ in Layer 2 is constructed (Figure 3a). In the following, the analytic solution will be derived within the framework of the Hamiltonian system via the solution procedure of the SSM in Figure 3, where two subproblems are given to establish the equivalence to the general problem. The basic systems of the two subproblems are both within a rectangular domain with zero heat flux, i.e., adiabatic BCs, on opposite edges at x=0 and x=a.

In Layer 1 (Figure 3b), the heat flux BCs should be satisfied: ${\left(\partial \bar{T}/\partial x\right)|}_{x=0}={\left(\partial \bar{T}/\partial x\right)|}_{x=a}=0$, which yields $B_{1}=0$ and $\sin\left(a\beta^{1}\right)=0$, leading to

(17) $$\begin{array}{c} \beta_{m}^{1}=\frac{m\pi }{a}\\ \beta_{-m}^{1}=-\frac{m\pi }{a} \end{array}\left(m=1,2,3,\cdots \right)$$

The eigenvalues are thus

(18) $$\mu_{\pm m}^{1}=\pm \sqrt{{\left(\beta_{m}^{1}\right)}^{2}+\frac{s}{\alpha_{1}}}$$

The corresponding eigenvectors are

(19) $$\begin{array}{c} X_{m}^{1}\left(x\right)=\cos\left(\beta_{m}^{1}x\right)\left[\begin{array}{c} 1\\ k_{1}\mu_{m}^{1} \end{array}\right]\\ X_{-m}^{1}\left(x\right)=\cos\left(\beta_{m}^{1}x\right)\left[\begin{array}{c} 1\\ -k_{1}\mu_{m}^{1} \end{array}\right] \end{array}$$

It is necessary to mention that $X_{m}^{1}\left(x\right)$ and $X_{-m}^{1}\left(x\right)$ are symplectically conjugated (i.e., $\int_{0}^{a}{\left[X_{m}^{1}\left(x\right)\right]}^{T}JX_{m}^{1}\left(x\right)dx\neq 0$), while any other combinations of two eigenvectors are symplectically orthogonal [22]. Moreover, we have constant eigenvalues $\mu_{01}^{1}=\sqrt{s/\alpha_{1}}$ and $\mu_{02}^{1}=-\sqrt{s/\alpha_{1}}$ from Equation (18) when n=0; thus, the corresponding eigenvectors are

(20) $$\begin{array}{l} X_{01}^{1}\left(x\right)=\left[\begin{array}{c} 1\\ k_{1}\mu_{01}^{1} \end{array}\right]\\ X_{02}^{1}\left(x\right)=\left[\begin{array}{c} 1\\ -k_{1}\mu_{01}^{1} \end{array}\right] \end{array}$$

Since all eigenvectors have been obtained for the Layer 1 problem, the state vector can be expanded according to the symplectic orthogonality and conjugacy, yielding

(21) $$Z_{1}=X^{1}\left(x\right)Y^{1}\left(y\right)$$

where

(22) $$\begin{array}{l} X^{1}\left(x\right)=\left[\cdots ,X_{01}^{1}\left(x\right),X_{m}^{1}\left(x\right),\cdots ,X_{02}^{1}\left(x\right),X_{-m}^{1}\left(x\right),\cdots \right]\\ Y^{1}\left(y\right)={\left[\cdots ,Y_{01}^{1}\left(y\right),Y_{m}^{1}\left(y\right),\cdots ,Y_{02}^{1}\left(y\right),Y_{-m}^{1}\left(y\right),\cdots \right]}^{T} \end{array}$$

Substituting Equation (21) into Equation (10) yields

(23) $$X^{1}\left(x\right)\frac{dY^{1}\left(y\right)}{dy}=H_{1}X^{1}\left(x\right)Y^{1}\left(y\right)+{\left[0,-{\bar{g}}_{1}\right]}^{T}$$

From the second of Equation (12), we have

(24) $$H_{1}X^{1}\left(x\right)=X^{1}\left(x\right)M^{1}$$

where $M^{1}=\mathrm{diag}\left(\cdots ,\mu_{01}^{1},\mu_{m}^{1},\cdots ,\mu_{02}^{1},\mu_{-m}^{1},\cdots \right)$. The vector concerning the heat source can be expanded by the symplectic eigenvectors, i.e.,

(25) $${\left[0,-{\bar{g}}_{1}\right]}^{T}=X^{1}\left(x\right)G^{1}$$

where $G^{1}={\left[\cdots ,g_{01}^{1},g_{m}^{1},\cdots ,g_{02}^{1},g_{-m}^{1},\cdots \right]}^{T}$ is the column matrix of the expansion coefficients. Taking Equations (24) and (25) into Equation (23), we have

(26) $$\frac{dY^{1}\left(y\right)}{dy}-M^{1}Y^{1}=G^{1}$$

i.e.,

(27) $$\begin{array}{c} \frac{dY_{01}^{1}\left(y\right)}{dy}-\mu_{01}^{1}Y_{01}^{1}\left(y\right)=g_{01}^{1},\frac{dY_{02}^{1}\left(y\right)}{dy}-\mu_{02}^{1}Y_{02}^{1}\left(y\right)=g_{02}^{1}\\ \frac{dY_{m}^{1}\left(y\right)}{dy}-\mu_{m}^{1}Y_{m}^{1}\left(y\right)=g_{m}^{1},\frac{dY_{-m}^{1}\left(y\right)}{dy}-\mu_{-m}^{1}Y_{-m}^{1}\left(y\right)=g_{-m}^{1} \end{array}$$

Multiplying both sides of Equation (25) by $X^{1}{\left(x\right)}^{T}J$ with integration concerning *x* over [0,a], i.e.,

(28) $$\int_{0}^{a}X^{1}{\left(x\right)}^{T}JX^{1}\left(x\right)G^{1}dx=\int_{0}^{a}X^{1}{\left(x\right)}^{T}{\left[0,-{\bar{g}}_{1}\right]}^{T}dx$$

we obtain

(29) $$\begin{array}{l} g_{01}^{1}=-\frac{Q_{1}}{2sak_{1}\mu_{01}^{1}}\delta \left(y-y_{1}\right),\;g_{m}^{1}=-\frac{Q_{1}}{sak_{1}\mu_{m}^{1}}\cos\left(\frac{m\pi x_{1}}{a}\right)\delta \left(y-y_{1}\right)\\ g_{02}^{1}=-\frac{Q_{1}}{2sak_{1}\mu_{02}^{1}}\delta \left(y-y_{1}\right),\;g_{-m}^{1}=-\frac{Q_{1}}{sak_{1}\mu_{-m}^{1}}\cos\left(\frac{m\pi x_{1}}{a}\right)\delta \left(y-y_{1}\right) \end{array}$$

Substituting Equation (29) into Equation (27), we obtain

(30) $$\begin{array}{c} Y_{01}^{1}\left(y\right)=C_{0}^{1}\exp\left(\mu_{01}^{1}y\right)-\frac{Q_{1}}{2sak_{1}\mu_{01}^{1}}H\left(y-y_{1}\right)\exp\left[\mu_{01}^{1}\left(y-y_{1}\right)\right]\\ Y_{02}^{1}\left(y\right)=D_{0}^{1}\exp\left(\mu_{02}^{1}y\right)-\frac{Q_{1}}{2sak_{1}\mu_{02}^{1}}H\left(y-y_{1}\right)\exp\left[\mu_{02}^{1}\left(y-y_{1}\right)\right]\\ Y_{m}^{1}\left(y\right)=C_{m}^{1}\exp\left(\mu_{m}^{1}y\right)-\frac{Q_{1}}{sak_{1}\mu_{m}^{1}}\cos\left(\frac{m\pi x_{1}}{a}\right)H\left(y-y_{1}\right)\exp\left[\mu_{m}^{1}\left(y-y_{1}\right)\right]\\ Y_{-m}^{1}\left(y\right)=D_{m}^{1}\exp\left(\mu_{-m}^{1}y\right)-\frac{Q_{1}}{sak_{1}\mu_{-m}^{1}}\cos\left(\frac{m\pi x_{1}}{a}\right)H\left(y-y_{1}\right)\exp\left[\mu_{-m}^{1}\left(y-y_{1}\right)\right] \end{array}$$

where $C_{0}^{1}$, $D_{0}^{1}$, $C_{m}^{1}$, and $D_{m}^{1}$ are undetermined coefficients and H is the Heaviside function. With the BCs at the bottom surface attached to human skin and the interface between Layer 1 and Layer 2:

(31) $$\begin{array}{c} {\bar{T}|}_{y=0}={\bar{T}}_{\mathrm{skin}}=\frac{T_{\mathrm{skin}}}{s}\\ {k_{1}\frac{\partial \bar{T}}{\partial y}|}_{y=b_{1}}=E_{0}+\sum_{m=1,2,3,\cdots }^{}E_{m}\cos\frac{m\pi x}{a} \end{array}$$

the temperature solution in Layer 1 (Figure 3b) in the Laplace transform domain is obtained as

(32) $$\begin{array}{l} \frac{k_{1}{\bar{T}}_{\mathrm{Layer}\;1}\left(x,y,s\right)}{Q_{1}}=\sum_{m=1,2,3,\cdots }^{}\frac{1}{s\zeta_{1}\zeta_{1m}\left[1+\exp\left(2\xi_{1}\right)\right]}\\ \times \exp\left(-\xi_{1}\hat{y}\right)\{\zeta_{1m}\exp\xi_{1}\left(s\theta_{1}+\delta_{1}\zeta_{1}\exp\xi_{1}\right)+\zeta_{1m}\exp\left(2\xi_{1}\hat{y}\right)\left(\delta_{1}\zeta_{1}-s\theta_{1}\exp\xi_{1}\right)\\ -\exp\xi_{1}H\left(b_{1}-y_{1}\right)\{\zeta_{1m}\cosh\left[\xi_{1}\left(1-{\hat{y}}_{1}\right)\right]\left[1-\exp\left(2\xi_{1}\hat{y}\right)\right]-4\zeta_{1}\cosh\xi_{1}\\ \times \sec h\xi_{1m}\exp\left(\xi_{1}\hat{y}\right)\cos\left(m\pi \hat{x}\right)\cos\left(m\pi {\hat{x}}_{1}\right)\sinh\left(\xi_{1m}\hat{y}\right)\cosh\left[\xi_{1m}\left(1-{\hat{y}}_{1}\right)\right]\}\\ -2\cosh\xi_{1}\exp\left[\xi_{1}\left(1+\hat{y}\right)\right]\{s\zeta_{1}\theta_{1m}\sec h\xi_{1m}\cos\left(m\pi \hat{x}\right)\sinh\left(\xi_{1m}\hat{y}\right)+H\left(y-y_{1}\right)\\ \times \left\{\zeta_{1m}\sinh\left[\xi_{1}\left(\hat{y}-{\hat{y}}_{1}\right)\right]+2\zeta_{1}\cos\left(m\pi \hat{x}\right)\cos\left(m\pi {\hat{x}}_{1}\right)\sinh\left[\xi_{1m}\left(\hat{y}-{\hat{y}}_{1}\right)\right]\right\}\}\} \end{array}$$

where $\hat{x}=x/a$, $\hat{y}=y/b_{1}$, ${\hat{x}}_{1}=x_{1}/a$, ${\hat{y}}_{1}=y_{1}/b_{1}$, $\xi_{1m}=b_{1}\mu_{m}^{1}$, $\zeta_{1m}=a\mu_{m}^{1}$, $\xi_{1}=b_{1}\mu_{01}^{1}$, $\zeta_{1}=a\mu_{01}^{1}$, $\delta_{1}=k_{1}T_{\mathrm{skin}}/Q_{1}$, $\theta_{1}=aE_{0}/Q_{1}$, and $\theta_{1m}=aE_{m}/Q_{1}$.

Following the same logic as described above, with the BCs at the top surface exposed to air and the interface between Layer 1 and Layer 2:

(33) $$\begin{array}{c} {\bar{T}|}_{y=b_{1}+b_{2}}={\bar{T}}_{\mathrm{surface}}=\frac{T_{\mathrm{surface}}}{s}\\ {k_{2}\frac{\partial \bar{T}}{\partial y}|}_{y=b_{1}}=E_{0}+\sum_{m=1,2,3,\cdots }^{}E_{m}\cos\frac{m\pi x}{a} \end{array}$$

the temperature solution in Layer 2 (Figure 3c) in the Laplace transform domain is obtained as

(34) $$\begin{array}{l} \frac{k_{2}{\bar{T}}_{\mathrm{Layer}\;2}\left(x,y,s\right)}{Q_{2}}=\sum_{m=1,2,3,\cdots }^{}\frac{1}{s\zeta_{1}\zeta_{1m}\left[1+\exp\left(2\xi_{2}\right)\right]\left[1+\exp\left(2\xi_{2m}\right)\right]}\{4\exp\left(\xi_{2}+\xi_{2m}\right)\\ \times \{\zeta_{1m}\cosh\xi_{2m}\left\{\delta_{2}\zeta_{1}\cosh\left[\xi_{1}\left(1-\hat{y}\right)\right]-s\theta_{2}\sinh\left[\xi_{2}+\xi_{1}\left(1-\hat{y}\right)\right]\right\}-s\zeta_{1}\theta_{2m}\cosh\xi_{2}\\ \times \cos\left(m\pi \hat{x}\right)\sinh\left[\xi_{2m}+\xi_{1m}\left(1-\hat{y}\right)\right]+\zeta_{1m}\cosh\xi_{2m}\cosh\left[\xi_{1}\left(1-\hat{y}\right)\right]H\left(b_{1}+b_{2}-y_{2}\right)\\ \times \sinh\left[\xi_{1}+\xi_{2}\left(1-{\hat{y}}_{2}\right)\right]\}-\left[1+\exp\left(2\xi_{2}\right)\right]\{\zeta_{1m}H\left(y-y_{2}\right)\sinh\left(\xi_{1}\hat{y}-\xi_{2}{\hat{y}}_{2}\right)\\ \times \left[1+\exp\left(2\xi_{2m}\right)\right]-4\zeta_{1}\exp\xi_{2m}\cos\left(m\pi \hat{x}\right)\cos\left(m\pi {\hat{x}}_{2}\right)\{\cosh\xi_{2m}H\left(y-y_{2}\right)\\ \times \sinh\left(\xi_{2m}{\hat{y}}_{2}-\xi_{1m}\hat{y}\right)+\cosh\left[\xi_{1m}\left(1-\hat{y}\right)\right]H\left(b_{1}+b_{2}-y_{2}\right)\sinh\left[\xi_{1m}+\xi_{2m}\left(1-{\hat{y}}_{2}\right)\right]\}\}\} \end{array}$$

where ${\hat{x}}_{2}=x_{2}/a$, ${\hat{y}}_{2}=y_{2}/b_{2}$, $\xi_{2m}=b_{2}\mu_{m}^{1}$, $\xi_{2}=b_{2}\mu_{01}^{1}$, $\delta_{2}=k_{2}T_{\mathrm{surface}}/Q_{2}$, $\theta_{2}=aE_{0}/Q_{2}$, and $\theta_{2m}=aE_{m}/Q_{2}$.

Both the temperature and heat flux should be continuous at the interface at $y=b_{1}$. The continuity of heat flux at $y=b_{1}$ has been satisfied, as revealed in Equations (31) and (33) where the same heat flux expression holds for Layer 1 and Layer 2. The continuity of temperature requires

(35) $${\bar{T}}_{\mathrm{Layer}\;1}\left(x,b_{1},s\right)={\bar{T}}_{\mathrm{Layer}\;2}\left(x,b_{1},s\right)$$

By Equation (35), the coefficients $E_{0}$ and $E_{m}\;\left(m=1,2,3,\cdots \right)$ are determined, and the analytic solution can be eventually given in the Laplace transform domain as

(36) $${\bar{T}}_{\mathrm{FEH}}\left(x,y,s\right)=\{\begin{array}{c} {\bar{T}}_{\mathrm{Layer}\;1}\left(x,y,s\right),0\leq y\leq b_{1}\\ {\bar{T}}_{\mathrm{Layer}\;2}\left(x,y,s\right),b_{1}\leq y\leq b_{1}+b_{2} \end{array}$$

It should be pointed out that an explicit expression of the inverse Laplace transform is strikingly complicated, and thus, it is not presented here.

## 4. Comprehensive Benchmark Results

To provide comprehensive numerical and graphical results as benchmarks for further structural designs, the analytic transient heat conduction results of bi-layered FEHs are given by the SSM with verification by the FEM. The considered circumstances are given with the following parameters: a=3 mm, $b_{1}=2\;\mathrm{mm}$, $b_{2}=1\;\mathrm{mm}$, $\alpha_{1}=2{\;\mathrm{mm}}^{2}/s$, $k_{1}=2\;W/\left(m\cdot K\right)$, $\rho_{1}c_{1}=10^{6}\;J/\left(m^{3}\cdot K\right)$, $\alpha_{2}=1\;{\mathrm{mm}}^{2}/s$, $k_{2}=1\;W/\left(m\cdot K\right)$, and $\rho_{2}c_{2}=10^{6}\;J/\left(m^{3}\cdot K\right)$. The BCs for Case 1 are set, with the top surface subjected to room temperature $T_{\mathrm{surface}}=25\;{}^{\circ}C$ and the bottom surface subjected to body temperature $T_{\mathrm{skin}}=37\;{}^{\circ}C$. The point heat source corresponds to $Q_{1}=0$ and $Q_{2}=3\;\mathrm{mW}/{\mathrm{mm}}^{3}$ at $\left(0,b_{1}\right)$. The BCs for Case 2 are given with the top and bottom surfaces subjected to $T_{\mathrm{skin}}=T_{\mathrm{surface}}=37\;{}^{\circ}C$, and the point heat source corresponds to $Q_{1}=0$ and $Q_{2}=10\;\mathrm{mW}/{\mathrm{mm}}^{3}$. The FEM is used via the software package ABAQUS with the four-node linear heat transfer quadrilateral shell element DC2D4 and the uniform mesh size of *a*/100 to achieve the convergent numerical solutions.

As revealed by the temperature results marked in bold in Table 1, rapid convergence is achieved such that dozens of series terms (20 at most) are enough to realize the accuracy to the last significant digit of five, and these results are justified by comparison with those by the FEM due to the lack of available analytic solutions. Therefore, 20 series terms are taken throughout this study.

The temperature results at typical locations within a broad time range are tabulated in Table 2 and Table 3. The comparison shows that all the present results agree quite well with those by the FEM, which confirms the validity and accuracy of the SSM. 

**Figure 4:**
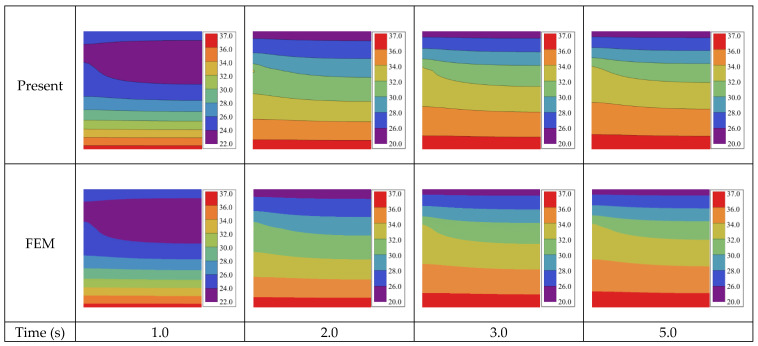
Temperature distribution (°C) for Case 1 on bi-layered transient heat conduction with a point heat source.

**Figure 5:**
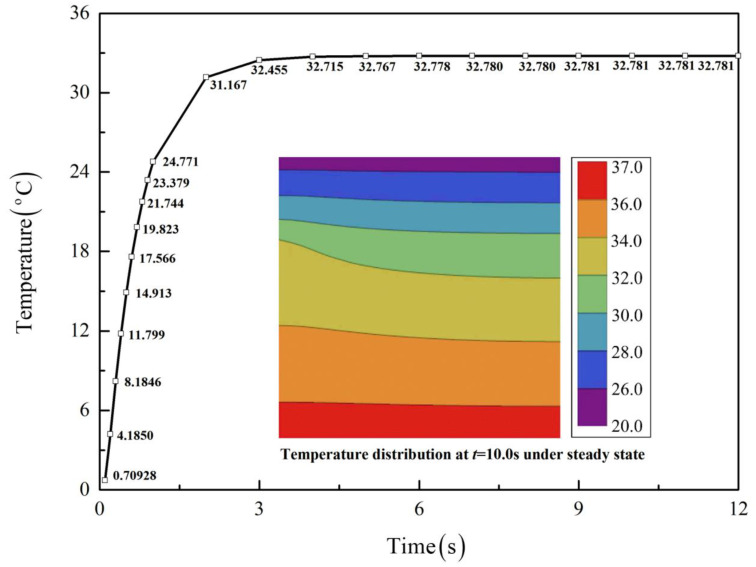
Time-dependent temperatures for Case 1 at (*a*/2, *a*/2), with the inset showing the temperature distribution under steady state.

To give more intuitive results, Figure 4 plots the contours of temperature distribution at different times for a more realistic Case 1 followed by comparison with the FEM, where satisfactory agreement is also observed. In addition, the time-dependent temperatures at a typical location, (*a*/2, *a*/2), are plotted in Figure 5, with the inset showing the temperature distribution under steady state when t > 9.0 s.

**Figure 6:**
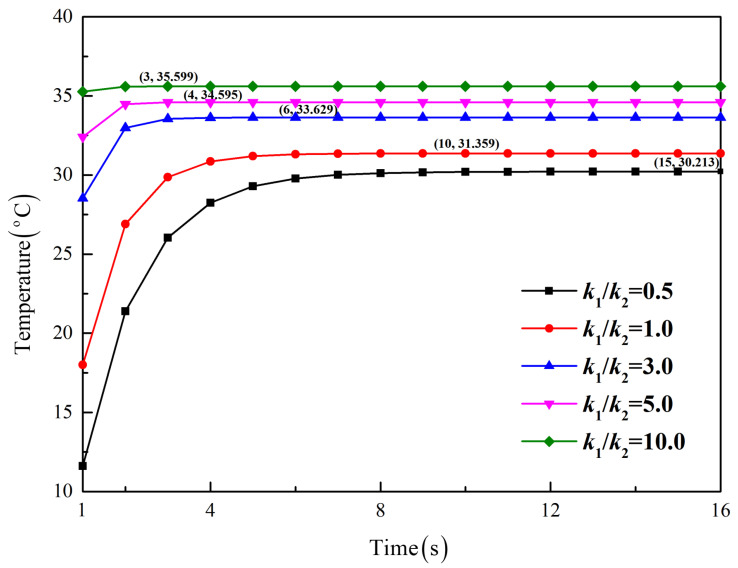
Influence of the thermal conductivity ratio on time-dependent temperatures for Case 1 at (*a*/2, *a*/2).

To further study the influence of thermal properties on the heat conduction behavior, a parameter analysis for Case 1 is conducted by changing the thermal conductivity $k_{1}$ but keeping $k_{2}$ constant, as shown in Figure 6. It is found that the larger the thermal conductivity ratio $k_{1}/k_{2}$ is, the faster the steady state is reached, which indicates a speed-up of thermotherapy. On the other hand, a larger $k_{1}/k_{2}$ corresponds to higher temperatures in Layer 1, which helps raise the temperature near human skin.

## 5. Conclusions

In this study, an analytic model of transient heat conduction by symplectic superposition is established for bi-layered FEHs and is well verified by the FEM modeling. By converting the problems in the Laplace transform domain into the Hamiltonian system, the mathematical techniques such as separation of variables and symplectic eigen expansion become available for deriving some new analytic solutions. The high accuracy and rapid convergence of the SSM are well confirmed by the numerical results as compared with the FEM results shown in tables and figures. The time-dependent temperature results, including the steady-state results, and the effect of thermal conductivity ratio on heat conduction have also been investigated. The developed analytic model provides a theoretical basis for transient heat conduction analysis of bi-layered FEHs, and it may be extended to more complicated problems, such as those of multi-layered FEHs and orthotropic FEHs. It is also worth mentioning that although the present solution does not involve Robin-type BCs, the SSM may be extended to heat conduction problems under such BCs as well.

## Figures and Tables

**Table 1:** Convergence study for the present temperature solutions (°C) at typical locations when *t* = 15.0 s.

Case	Location	Number of Series Terms					FEM
		5	10	20	30	50	
1	(*a*/6, *a*/6)	**35.699**	35.699	35.699	35.699	35.699	35.699
	(*a*/2, *a*/6)	**35.613**	35.613	35.613	35.613	35.613	35.613
	(5*a*/6, *a*/6)	**35.563**	35.563	35.563	35.563	35.563	35.563
	(*a*/6, *a*/2)	33.197	**33.190**	33.190	33.190	33.190	33.190
	(*a*/2, *a*/2)	32.784	**32.781**	32.781	32.781	32.781	32.781
	(5*a*/6, *a*/2)	32.644	**32.641**	32.641	32.641	32.641	32.641
	(*a*/6, 5*a*/6)	28.499	**28.492**	28.492	28.492	28.492	28.492
	(*a*/2, 5*a*/6)	28.171	**28.167**	28.167	28.167	28.167	28.167
	(5*a*/6, 5*a*/6)	28.081	28.078	**28.077**	28.077	28.077	28.077
2	(*a*/6, *a*/6)	**37.662**	37.662	37.662	37.662	37.662	37.662
	(*a*/2, *a*/6)	**37.378**	37.378	37.378	37.378	37.378	37.378
	(5*a*/6, *a*/6)	**37.210**	37.210	37.210	37.210	37.210	37.210
	(*a*/6, *a*/2)	39.324	39.300	**39.301**	39.301	39.301	39.302
	(*a*/2, *a*/2)	37.947	**37.935**	37.935	37.935	37.935	37.935
	(5*a*/6, *a*/2)	37.479	37.469	**37.468**	37.468	37.468	37.468
	(*a*/6, 5*a*/6)	38.662	38.638	**38.639**	38.639	38.639	38.640
	(*a*/2, 5*a*/6)	37.570	37.557	**37.558**	37.558	37.558	37.557
	(5*a*/6, 5*a*/6)	37.269	37.259	**37.258**	37.258	37.258	37.258

**Table 2:** FEM-validated temperature solutions (^o^C) at different times for Case 1.

Time (s)	Method	Location								
		(*a*/6, *a*/6)	(*a*/2, *a*/6)	(5*a*/6, *a*/6)	(*a*/6, *a*/2)	(*a*/2, *a*/2)	(5*a*/6, *a*/2)	(*a*/6, 5*a*/6)	(*a*/2, 5*a*/6)	(5*a*/6, 5*a*/6)
0.5	Present	27.648	27.586	27.557	15.266	14.913	14.824	17.501	17.221	17.170
	FEM	27.580	27.519	27.490	15.150	14.799	14.712	17.443	17.165	17.114
1.0	Present	32.134	32.053	32.007	25.171	24.771	24.641	23.513	23.195	23.113
	FEM	32.088	32.007	31.961	25.068	24.669	24.539	23.450	23.133	23.051
1.5	Present	34.101	34.016	33.967	29.594	29.186	29.047	26.257	25.934	25.845
	FEM	34.070	33.986	33.936	29.524	29.116	28.978	26.214	25.891	25.802
2.0	Present	34.982	34.896	34.846	31.576	31.167	31.027	27.489	27.165	27.075
	FEM	34.963	34.878	34.828	31.535	31.125	30.986	27.463	27.139	27.049
2.5	Present	35.377	35.292	35.241	32.466	32.056	31.916	28.042	27.717	27.627
	FEM	35.366	35.281	35.231	32.443	32.033	31.893	28.027	27.703	27.613
3.0	Present	35.554	35.469	35.419	32.865	32.455	32.315	28.290	27.965	27.875
	FEM	35.548	35.463	35.413	32.853	32.443	32.303	28.282	27.957	27.868
5.0	Present	35.693	35.607	35.557	33.177	32.767	32.627	28.484	28.159	28.069
	FEM	35.692	35.607	35.557	33.176	32.767	32.626	28.483	28.159	28.069
7.5	Present	35.698	35.613	35.563	33.190	32.780	32.640	28.492	28.167	28.077
	FEM	35.698	35.613	35.563	33.190	32.780	32.640	28.492	28.167	28.077
10.0	Present	35.699	35.613	35.563	33.190	32.781	32.641	28.492	28.167	28.077
	FEM	35.699	35.613	35.563	33.190	32.781	32.641	28.492	28.167	28.077
15.0	Present	35.699	35.613	35.563	33.190	32.781	32.641	28.492	28.167	28.077
	FEM	35.699	35.613	35.563	33.190	32.781	32.641	28.492	28.167	28.077

**Table 3:** FEM-validated temperature solutions (°C) at different times for Case 2.

Time (s)	Method	Location								
		(*a*/6, *a*/6)	(*a*/2, *a*/6)	(5*a*/6, *a*/6)	(*a*/6, *a*/2)	(*a*/2, *a*/2)	(5*a*/6, *a*/2)	(*a*/6, 5*a*/6)	(*a*/2, 5*a*/6)	(5*a*/6, 5*a*/6)
0.5	Present	28.216	28.008	27.912	18.086	16.909	16.614	25.448	24.514	24.342
	FEM	28.147	27.941	27.847	17.953	16.781	16.490	25.359	24.432	24.262
1.0	Present	33.454	33.183	33.029	29.828	28.494	28.059	32.750	31.692	31.416
	FEM	33.399	33.130	32.976	29.706	28.373	27.940	32.674	31.618	31.344
1.5	Present	35.777	35.495	35.330	35.058	33.697	33.236	36.002	34.924	34.629
	FEM	35.740	35.459	35.294	34.976	33.615	33.155	35.951	34.873	34.579
2.0	Present	36.816	36.533	36.365	37.398	36.033	35.567	37.457	36.375	36.077
	FEM	36.794	36.511	36.344	37.349	35.984	35.518	37.426	36.345	36.046
2.5	Present	37.282	36.998	36.831	38.447	37.081	36.615	38.109	37.027	36.728
	FEM	37.270	36.986	36.819	38.420	37.053	36.587	38.092	37.010	36.711
3.0	Present	37.492	37.208	37.040	38.918	37.552	37.085	38.401	37.319	37.020
	FEM	37.485	37.201	37.033	38.903	37.537	37.070	38.392	37.310	37.011
5.0	Present	37.655	37.371	37.203	39.286	37.920	37.453	38.630	37.548	37.249
	FEM	37.655	37.370	37.203	39.285	37.919	37.452	38.629	37.547	37.248
7.5	Present	37.662	37.378	37.210	39.301	37.935	37.468	38.639	37.557	37.258
	FEM	37.662	37.378	37.210	39.301	37.935	37.468	38.640	37.557	37.258
10.0	Present	37.662	37.378	37.210	39.301	37.935	37.468	38.639	37.557	37.258
	FEM	37.662	37.378	37.210	39.302	37.935	37.468	38.640	37.557	37.258
15.0	Present	37.662	37.378	37.210	39.301	37.935	37.468	38.639	37.558	37.258
	FEM	37.662	37.378	37.210	39.302	37.935	37.468	38.640	37.557	37.258

## Data Availability

The data presented in this study are available on reasonable request from the corresponding author.
